# Deep sequencing of circulating tumor DNA detects molecular residual disease and predicts recurrence in gastric cancer

**DOI:** 10.1038/s41419-020-2531-z

**Published:** 2020-05-11

**Authors:** Jian Yang, Yuhua Gong, Vincent K. Lam, Yan Shi, Yanfang Guan, Yanyan Zhang, Liyan Ji, Yongsheng Chen, Yongliang Zhao, Feng Qian, Jun Chen, Pingang Li, Fan Zhang, Jiayin Wang, Xuanping Zhang, Ling Yang, Scott Kopetz, P. Andrew Futreal, Jianjun Zhang, Xin Yi, Xuefeng Xia, Peiwu Yu

**Affiliations:** 1 Department of General Surgery and Center of Minimal Invasive Gastrointestinal Surgery, The First Hospital Affiliated to Army Medical University, Chongqing, China; 2 Geneplus-Beijing, Beijing, China; 3 Department of Computer Science and Technology, School of Electronic and Information Engineering, Xi’an Jiaotong University, Xi’an, Shaanxi China; 4 Sidney Kimmel Comprehensive Cancer Center, Johns Hopkins University School of Medicine, Baltimore, MD USA; 5 Department of Gastrointestinal Medical Oncology, Division of Cancer Medicine, The University of Texas MD Anderson Cancer Center, Houston, TX USA; 6 Department of Genomic Medicine, The University of Texas MD Anderson Cancer Center, Houston, TX USA; 7 Department of Thoracic/Head and Neck Medical Oncology, The University of Texas MD Anderson Cancer Center, Houston, TX USA

**Keywords:** Gastric cancer, Predictive markers

## Abstract

Identifying locoregional gastric cancer patients who are at high risk for relapse after resection could facilitate early intervention. By detecting molecular residual disease (MRD), circulating tumor DNA (ctDNA) has been shown to predict post-operative relapse in several cancers. Here, we aim to evaluate MRD detection by ctDNA and its association with clinical outcome in resected gastric cancer. This prospective cohort study enrolled 46 patients with stage I–III gastric cancer that underwent resection with curative intent. Sixty resected tumor samples and 296 plasma samples were obtained for targeted deep sequencing and longitudinal ctDNA profiling. ctDNA detection was correlated with clinicopathologic features and post-operative disease-free (DFS) and overall survival (OS). ctDNA was detected in 45% of treatment-naïve plasma samples. Primary tumor extent (T stage) was independently associated with pre-operative ctDNA positivity (*p* = 0.006). All patients with detectable ctDNA in the immediate post-operative period eventually experienced recurrence. ctDNA positivity at any time during longitudinal post-operative follow-up was associated with worse DFS and OS (HR = 14.78, 95%CI, 7.991–61.29, *p* < 0.0001 and HR = 7.664, 95% CI, 2.916–21.06, *p* = 0.002, respectively), and preceded radiographic recurrence by a median of 6 months. In locoregional gastric cancer patients treated with curative intent, these results indicate that ctDNA-detected MRD identifies patients at high risk for recurrence and can facilitate novel treatment intensification studies in the adjuvant setting to improve survival.

## Introduction

Gastric cancer is one of the most common malignancies in the world (especially in East Asia) and is the third leading cause of cancer-related death^1,2^. Curative surgery remains the primary treatment choice for locoregional gastric cancer^3^. However, even with current adjuvant chemotherapy regimens such as oral S-1 and CAPOX^4,5^, clinical outcome remains poor with recurrence rates as high as 88% and 5-year overall survival of only about 20% for node-positive disease^3,6–9^. Most recurrences occur within 2 years after surgery and often involve advanced disease that can no longer be treated with curative intent.

Routine clinical imaging and biomarker modalities cannot reliably detect post-operative molecular residual disease (MRD) or micrometastatic recurrence. Commonly used serum tumor markers, including carcinoembryonic antigen (CEA) and cancer antigen 19-9 (CA 19-9), detect only about 40% of recurrences^9^ with poor sensitivity and specificity^10^. Circulating cell-free tumor DNA (ctDNA) is a promising biomarker for non-invasive molecular profiling, monitoring and predicting response to systemic treatment^11–13^, and more recently, cancer detection as well^14,15^. Previous studies have shown that ctDNA is a reliable biomarker for detecting MRD in breast cancer^16^, colon cancer^17^, and lung cancer^18,19^. In gastric cancer, recent studies have demonstrated the potential of using ctDNA for monitoring clinical response to immunotherapy^20^ and tracking anti-HER2 resistance^21^ in the metastatic setting. However, studies about the prognostic utility of ctDNA and clinical determinants of increased ctDNA shedding in gastric cancer are limited^22^. In this study, we sought to evaluate the utility of longitudinal ctDNA targeted deep sequencing for detecting MRD and micrometastatic recurrence in resected, locoregional gastric cancer.

## Materials and methods

### Study design

This was a prospective cohort study of patients with stage I–III, resectable gastric cancer enrolled at First Hospital Affiliated to Army Medical University in Chongqing, China, between 2015 and 2017. Eligible patients underwent gastrectomy with curative intent, followed by adjuvant chemotherapy (SOX) when indicated by standard of care clinical guidelines^23^. Blood samples were collected prior to surgery and at multiple time points thereafter during longitudinal follow-up. Follow-up occurred every 3–6 months in the first year after surgery, then every 6–12 months thereafter. Each follow-up assessment included physical examination, routine blood tests, serum tumor marker level assessment (e.g., CEA and CA19-9), gastroscopy, chest radiograph, and abdominal CT scan. The study was approved by the Research Ethics Committee of the First Hospital Affiliated to Army Medical University.

### Targeted sequencing analysis of tissue and plasma DNA

Tumor tissue was obtained for targeted deep sequencing from the resection specimen and, if available, at time of recurrence. Blood samples were obtained for sequencing analysis 1 month after surgery, then every 3 months for the first year, and every 6 months thereafter. Next generation sequencing of tissue and plasma specimens were performed as previously described^13,24^, with a targeted sequencing panel covering 1021 genes and total genomic region of 1.09 Mb (Supplementary Table 1). Peripheral blood mononuclear cells (PBMCs) were also sequenced as normal controls to minimize non-tumor related mutations such as germline mutations and mutations from clonal hematopoiesis.

For tissue specimens, first, we extracted genomic DNA from fresh frozen or FFPE tissue specimens using a QIAamp DNA Mini Kit (Qiagen) and ReliaPrep™ FFPE gDNA Miniprep System (Promega), respectively, according to the manufacturer’s instructions. Genomic DNA was extracted from matched PBMC using a QIAamp DNA Mini Kit (Qiagen) according to the manufacturer’s instructions. After fragmentation with a Covaris S2 ultrasonicator (Covaris) to generate fragments with a 300-bp peak, we performed library construction reactions to generate sequencing libraries using NEBNext® Ultra™ DNA Library Prep Kit for Illumina® (NEB) according to the manufacturer’s instructions. Then, we enriched the library DNA for targeted regions using customized probe sets (Integrated DNA Technologies, IDT) according to manufacturer’s instructions. The enriched libraries were sequenced on an Illumina Hiseq 3000 sequencer to generate approximately 1 Gb, 2 Gb, and 3 Gb data for libraries from PBMCs, fresh frozen tissue specimens, and FFPE tissue specimens, respectively.

Mutect 2.0^25^ was used to call somatic single nucleotide variations and small insertions and deletions; copy number analysis for targeted resequencing (CONTRA)^26^ was used for identification of copy number alterations. We included probes that targeted known structure variation in all three probe sets and identified somatic structure variations using a local algorithm. In short, chimeric reads and discordant read pairs were identified to detect structure variations.

In total, we collected 60 tumors and excluded four during data quality control. The remaining 56 tumors, including 48 surgical resected primary tumors from 46 patients (there were two patients each with two primary tumor specimens collected), one original tumor from a patient with gastric remnant cancer, one regional and four distant recurrences, and two newly developed esophageal cancers. For the two patients with two primary tumors collected, we combined the data from different primary tumors within the same patient in the results.

For cell-free DNA samples, we performed targeted deep sequencing to identify somatic variations with low abundance, as previously described^24^. Briefly, we extracted DNA from plasma samples using the QIAsymphony DSP Circulating DNA Kit (Qiagen) and constructed a library using NEBNext® Ultra™ DNA Library Prep Kit for Illumina® (NEB) according to the manufacturers’ instruction. Sequencing adapters with a unique identifier tag were added to DNA fragments during library construction. We performed genomic region enrichment using same probe sets as for the tumor DNA. DNA sequencing were performed using an Illumina Hiseq 3000 to generate about 15 Gb of data for each sample.

A local pipeline was used to identify somatic variants in ctDNA after filtering out germline variants using PBMC DNA. For variants identified in the matched tumor DNA, we tracked reads carrying the same variants in ctDNA and regarded those supported by two or more reads as being present. We defined ctDNA detection as the detection of one or more mutations.

In total, 296 blood samples were donated; three of these were excluded due to failed sequencing and three were excluded as snp data not matched with the germline DNA.

### Statistical analysis

We assessed differences in clinical characteristics between pre-operative ctDNA-positive and ctDNA-negative patients using Fisher’s exact test for categorical variables and Mann-Whitney (rank sum) test for continuous variables. Correlation between ctDNA maximum VAF and tumor size or tumor volume were assessed by Spearman correlation. In the multivariate analyses, univariate factors with a *p* value < 0.1 were included. Continuous independent variables were found to be linearly related to the logit of the dependent variable (Box-Tidwell procedure). We assessed the association between ctDNA detection and disease-free survival (DFS) and overall survival (OS) by the log-rank method. All statistical tests were two-sided and *p* values <0.05 were considered significant. Unless otherwise specified, SPSS (version 23.0; Armonk, NY, IBM Corp) and GraphPad Prism (version 6.0c) were used for all analyses.

## Results

### Pre-operative primary tumor genomic profile and concordance with ctDNA

Clinicopathological characteristics of all 46 enrolled patients are shown in Table 1 and Supplementary Table 2. Half of the patients had stage III disease. Almost all patients were treated with adjuvant chemotherapy (45/46, 98%) and two patients had neoadjuvant chemotherapy. Targeted sequencing of pre-operative tumor tissue was performed at an average depth of 880×. As shown in Supplementary Fig. 1 and Supplementary Table 3, somatic mutations were detected in 45 of the 46 patients’ primary tumors with a median of three mutations per patient (range: 1–21 mutations). Consistent with known somatic landscape of gastric adenocarcinoma, *TP53* was the most frequently mutated gene (20/46, 43%)^27^. *ERBB2* amplification was observed in two tumors, detected by copy number variation and immunohistochemistry. To assess the feasibility of genomic profiling of gastric cancer using ctDNA, somatic mutations from 44 patients with matched pre-operative tissue and plasma were compared. A median of 50% (17–100%) of mutations detected in the tissue DNA were also detected in paired ctDNA samples in 19 patients, suggesting strong overall concordance (Supplementary Fig. 2).

**Table 1:** Clinical characteristics.

Variable	All (*n* = 46)
*Age, years*	
Median	54
Range	28-78
Sex, n (%)	
Female	8 (17)
Male	38 (83)
*Tumor site, n (%)*	
Cardia	8 (17)
Body	18 (39)
Antrum	18 (39)
Diffuse	2 (4)
*Stage, n (%)*	
I	9 (20)
II	12 (26)
III	23 (50)
NA	2 (4)
*Lauran classification, n (%)*	
Intestinal	12 (26)
Diffuse	13 (28)
Mixed	20 (43)
Indeterminate	1 (2)
*Early/Advanced, n (%)*	
Early	7 (15)
Advanced	39 (85)
*Borrmann classification, n (%)*	
Type II	11 (24)
Type III	25 (54)
Type IV	3 (7)
*Tumor differentiation, n (%)*	
Well	1 (2)
Moderate	9 (20)
Poor	36 (78)
*Helicobacter pylori infection, n (%)*	
Positive	100 (100)
Negative	0 (0)
*Recurrence, n (%)*	
Yes	19 (41)
No	25 (54)
NA	2 (4)

### Genomic profiling of gastric cancer from pre-operative ctDNA

The ctDNA genomic landscape of gastric cancer was delineated from analysis of somatic mutations, copy number alterations, and structural variants in these pre-operative plasma samples. In the 44 samples analyzed, mutations were detected in 20 samples (45%) with maximum VAF ranging from 0.1% to 31.18%. Consistent with the tissue genomic profile, *TP53* was the most frequently mutated gene in ctDNA (Supplementary Fig. 3). Notably, an instance of *CLDN18-ARHGAP26* fusion was also detected (and confirmed in matched tissue profiling), highlighting the ability of the ctDNA assay to detect structural variants.

### Determinants of ctDNA shedding in resectable gastric cancer

**Fig. 1:**
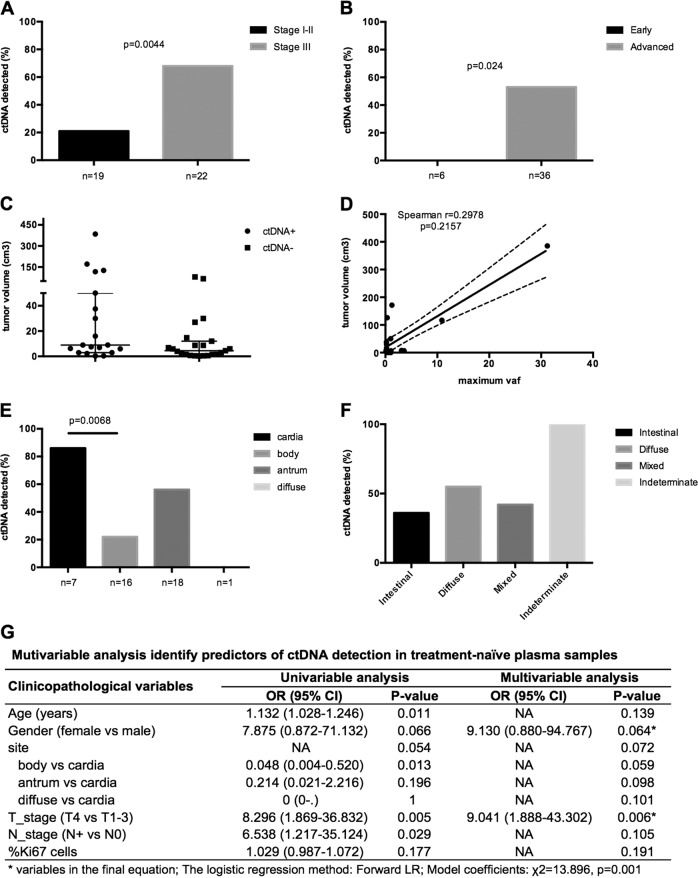
Clinical determinates of ctDNA detection in gastric cancer. Fractions of cases with ctDNA detected were shown in gastric cancer groups with different AJCC/UICC stage (**a**), early/advanced stage (**b**), tumor site (**e**) and histologic Lauren classification (**f**). Differences were assessed using Fisher’s Exact test, and *p* value were shown when less than 0.05. **c** tumor volume of cases with ctDNA detected (ctDNA+) or not (ctDNA-) were shown. The line indicates median with interquartile range. **d** Correlation with maximum VAF of cell-free DNA mutations and tumor volume were shown, the line indicates best fit values and 95% confidence intervals of linear regression. **g** Multivariable analysis results were shown.

To identify potential determinates of ctDNA shedding in gastric cancer patients, we compared ctDNA detection rates between patients grouped by various clinicopathologic features (Fig. 1 and Supplementary Table 4). As expected, pre-operative ctDNA positivity was associated with disease stage; 68% (15 of 22) of stage III cases were ctDNA positive, compared with 21% (4 of 19) of stage I and II cases (*p* = 0.0044, Fig. 1a). No patients with early gastric carcinoma (gastric adenocarcinoma confined to the mucosa and submucosa of the stomach, with or without regional lymph node metastases) had detectable pre-operative ctDNA (*p* = 0.024, Fig. 1b). Patients with a higher T stage or lymph node involvement were more likely to have detectable ctDNA (*p* = 0.005 and *p* = 0.029, respectively). In addition, there was a trend towards a positive association between tumor volume and ctDNA-positivity (median 9 cm^3^ in ctDNA mutation positive group versus 4.5 cm^3^ in ctDNA mutation negative group, *p* = 0.0582, Fig. 1c), though no specific correlation with maximum VAF was observed (Fig. 1d). Interestingly, primary tumors located in the gastric cardia appeared to have increased ctDNA levels compared to those located in the gastric body (*p* = 0.007, Fig. 1e) and non-cardia tumors (*p* = 0.034). No association of ctDNA status and Lauren classification was observed (Fig. 1f). In the multivariable analysis, only T stage remained a significant predictor of ctDNA shedding after accounting for age, gender, tumor site, lymph node status, and Ki67 proliferation index (*p* = 0.006, Fig. 1g).

### Post-operative ctDNA detects clinically significant MRD

**Fig. 2:**
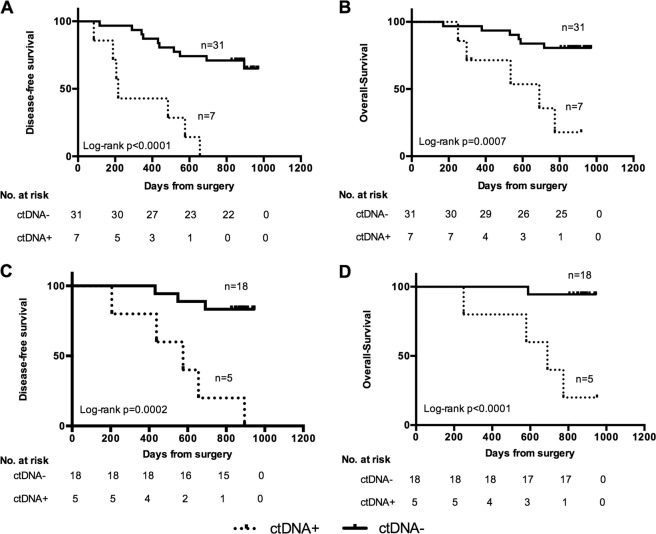
Patient survival is associated with ctDNA detection results. Kaplan–Meier curves for disease-free survival data in relation to ctDNA detection in Plasma obtained after surgery before initiation of adjuvant treatment (**a**) and after completion of adjuvant treatment (**c**). **b**, **d** show Kaplan–Meier curves of overall survival data in relation to ctDNA detection in the same samples as disease-free survival curves. ctDNA+: ctDNA was detected; ctDNA−: ctDNA was not detected. The number of patients in each group and the log-rank *p* value are shown.

We next assessed whether ctDNA positivity after surgery correlated with eventual tumor recurrence, suggesting the presence of MRD. Post-operative samples (collected prior to any adjuvant chemotherapy; 9–48 days after surgery) showed that ctDNA was detected in 18% (7 of 38) of evaluable patients (median maximum VAF of mutation is 0.23%, range 0.11–2.15%). ctDNA positivity after surgery was strongly associated with increased risk of relapse (100% recurrence in positive group vs 32% in negative group, p = 0.0015, Fisher’s exact test) and worse DFS (*p* < 0.0001, HR = 6.56, 95% CI, 8.316–208.5) and OS (*p* = 0.0007, HR = 5.959, 95% CI, 3.765–138.1) (Fig. 2a, b). The median DFS in patients with and without detectable post-operative ctDNA was 216 days and not reached, respectively. In multivariable analysis accounting for T stage and tumor site, post-operative ctDNA positivity remained an independent predictor of recurrence (*p* = 0.005, Supplementary Table 5). The sensitivity and specificity of post-operative ctDNA positivity in predicting recurrence at various time points is shown in Supplementary Table 6. The sensitivity and specificity of post-operative ctDNA positivity in predicting recurrence at 30 months were 39% and 100%, respectively. ctDNA positivity after completion of adjuvant chemotherapy was also similarly associated with worse DFS and OS (Fig. 2c, d). Taken together, these findings demonstrate that ctDNA-detected MRD after definitive therapy in resectable gastric cancer identifies patients at high risk for worse clinical outcome.

### Longitudinal ctDNA profiling enables early detection of recurrence

**Fig. 3:**
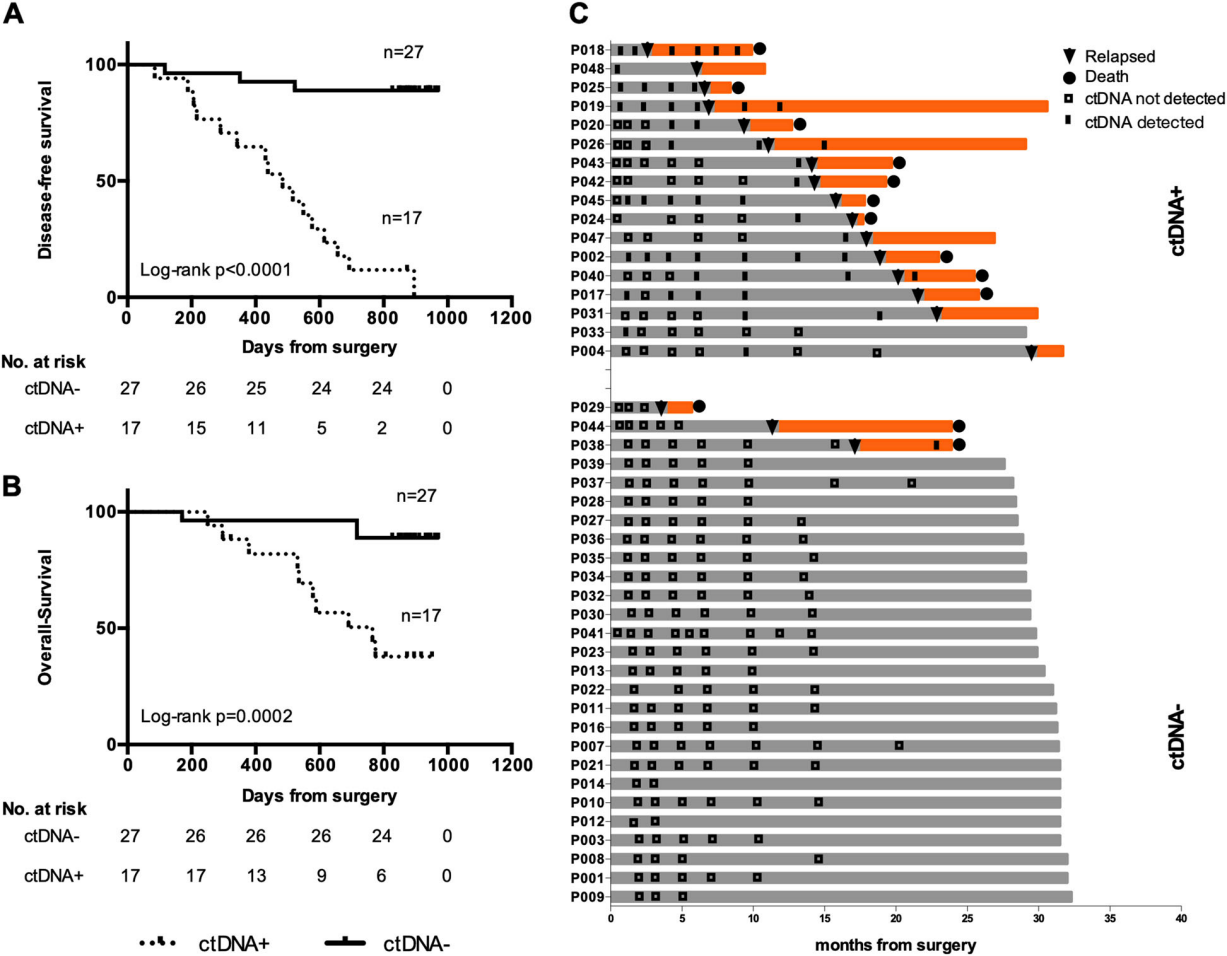
Mutation tracking at serial time points predict patient survival. Kaplan–Meier curves of disease-free survival (**a**) and overall survival (**b**) associated with detection of ctDNA in any post-operative plasma samples in patients with gastric cancer resected. Numbers of patients and log-rank *p* value are shown. **c** Detailed survival data of patients are shown, with gray bars indicating disease-free survival and orange bars indicating survival after recurrence. ctDNA+: ctDNA was detected; ctDNA−: ctDNA was not detected.

Using plasma samples collected at post-operative longitudinal time points as described earlier, we next assessed the utility of ctDNA as biomarker for post-surgical disease monitoring. Median follow-up time was 29.1 months (range 5.7–32.3 months). Detection of ctDNA mutations at any of these post-operative time points was associated with worse DFS (*p* < 0.0001) and OS (*p* = 0.0002) (Fig. 3). Of patients with recurrence, 41% (7 of 17) of patients had detectable ctDNA in the first post-operative sample and 84% (16 of 19) had ctDNA detected in at least one post-operative sample. In patients without recurrence, 100% (21 of 21) and 96% (24 of 25) had no detectable ctDNA at the first post-operative time point and any post-operative time point, respectively. In patients with detectable ctDNA at the post-operative, post-adjuvant chemotherapy, and subsequent longitudinal time points, the incidence of recurrence was 100% (7 of 7), 100% (5 of 5), and 94% (16 of 17), respectively. Among the three patients that recurred without any post-operative ctDNA positivity (P029, P038, P044), blood samples were actually not available for P029 and P044 at the time of recurrence. For patient P038, whole exome sequencing (WES) of the primary tumor revealed clonal mutations that were not included in the targeted sequencing panel and thus recurrence was not detected (Supplementary Fig. 4).

**Fig. 4:**
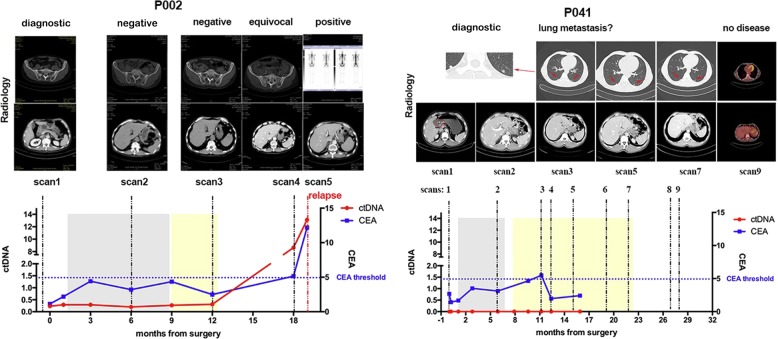
Potential application of ctDNA detection in post-surgery surveillance for patients with gastric cancer. Detailed radiology results, ctDNA and CEA changes for patient P002 and P041 are shown. Dynamic changes of ctDNA (red line) and CEA (blue line), adjuvant chemotherapy with SOX regimen (gray box) and S-1 (yellow box) are shown at bottom panels. Representative radiology images are shown, including abdominal and pelvic CT scan, chest CT scan, PET-CT, and bone scan.

ctDNA was detected a median of 179 days prior to radiographic recurrence (Supplementary Figs. 5 and 6). Furthermore, ctDNA was detected in 29% (10 of 34) of radiographic time points that were considered radiologic equivocal for possible recurrence and all of these ctDNA positive patients ultimately recurred (ctDNA detected vs not detected: 100% vs 21%, *p* < 0.0001, Fisher’s exact test). Radiographic, CEA, and ctDNA findings are shown for two patients in Fig. 4 to illustrate how ctDNA can help clarify equivocal imaging and/or CEA findings to enhance disease monitoring accuracy in gastric cancer.

### Longitudinal ctDNA profiling enables profiling of clonal evolution

Dynamic changes of mutations were analyzed longitudinally in six patients with ctDNA detected after surgery (Supplementary Fig. 7). Twenty-one new mutations (not present in baseline ctDNA) were detected during or after the completion of chemotherapy; five of which were identified in the primary tumor, the rest of which were neither detected in the primary tumor nor in baseline ctDNA. No functional clustering were seen in these 16 new mutations, though they did include known driver genes such as *TP53*, *RB1*, *PIK3CA*, *ATR*. These results suggest the importance of broadly tracking ctDNA changes using a large gene panel instead of tumor-informed approach that focuses only on select tumor-derived mutations.

## Discussion

This prospective cohort study evaluated the clinical utility of ctDNA for detection of MRD and longitudinal disease monitoring in locoregional gastric cancer treated with curative intent. We found that ctDNA positivity in the immediate post-operative period (or any time thereafter during longitudinal follow-up) was significantly associated with worse DFS and OS, suggesting the presence of clinically significant MRD. Moreover, ctDNA positivity preceded radiographic recurrence by a median of 6 months. Similar to what has previously been reported in other solid tumors, our results demonstrate in gastric cancer for the first time that ctDNA is a sensitive and specific biomarker for identifying patients at high risk for recurrence after definitive therapy for locoregional disease.

As ctDNA assays continue to mature, the evidence establishing promising clinical potential of this non-invasive biomarker is rapidly growing. Key studies in breast^16^, colorectal^17,28^, and lung cancer^18,19^ have demonstrated that ctDNA profiling after definitive therapy can identify patients who have MRD and thus worse clinical outcome. By affirming that this ctDNA-directed MRD concept also applies in gastric cancer, our findings help provide clinical rationale for novel adjuvant gastric cancer clinical trials that can utilize MRD to better identify high-risk patients for treatment intensification (e.g. personalized neoantigen vaccines) and potentially leverage ctDNA clearance as a surrogate endpoint for survival^29^.

In our study, longitudinal post-operative ctDNA sampling also could identify gastric cancer patients at increased risk for recurrence, likely heralding the presence of micrometastatic (radiographic occult) disease. The high positive predictive value of ctDNA positivity for disease progression complements recent metastatic gastric cancer data showing that on-treatment changes in ctDNA levels correlate with response and survival^20^ and suggests that ctDNA can be a powerful adjunct to radiographic imaging (and non-specific serum biomarkers such as CEA) for disease monitoring in gastric cancer. Moreover, during or after the completion of chemotherapy, several new mutations were observed in ctDNA, including driver mutations in *TP53*, *RB1*, *PIK3CA* and *ATR* genes. This illustrates an advantage of ctDNA monitoring with a platform that is not dependent on a priori tissue genotyping.

As potential clinical applications increase for ctDNA in gastric cancer (especially ctDNA-directed trials) it is critical to further our understanding of tumor factors that contribute to ctDNA shedding heterogeneity. As expected, we saw that tumor extent (T stage) was an independent predictor of ctDNA shedding. Among other factors that have been shown to impact ctDNA levels in other cancer types (e.g. nodal status, histology, Ki-67, tumor volume), we also observed that nodal status, tumor volume, and gastric cardia tumors appear to correlate with increased ctDNA shedding. These findings require validation in larger gastric cancer data sets.

In keeping with other major ctDNA assays currently in clinical use or research use, our ctDNA platform demonstrated extremely high specificity for detecting disease progression. The challenging application of ctDNA for MRD and early recurrence detection also requires sufficient sensitivity. Our observed pre-operative ctDNA detection rate of 45% with minimum VAF of 0.1% is consistent with that of other ctDNA assays in the solid tumor MRD literature (sensitivity ranging from 33% to 57%)^16,28,30,31^. However, MRD for very early-stage, localized gastric cancer may be undetectable at this range of sensitivity. Also, more definitive conclusions about clinicopathologic determinants of pre-operative ctDNA shedding will require a larger study.

There are potential limitations to our study. For example, our estimation of ctDNA lead-time prior to radiographic recurrence may be limited by the relatively modest sample size of patients with recurrent gastric cancer and could also represent an over-estimation since CT scans were generally not performed at the same time as blood collection for ctDNA analysis.

In conclusion, we have demonstrated that a ctDNA assay that can detect MRD and monitor for disease recurrence in definitively-treated locoregional gastric cancer. ctDNA positivity at any post-operative time point was associated with significantly worse clinical outcome and preceded radiographic detection of recurrence with substantial lead-time. These data affirm ctDNA as an emerging clinical biomarker for disease monitoring in gastric cancer and solid tumors overall.

### Ethics approval and consent to participate

The study was approved by the Research Ethics Committee of the First Hospital Affiliated to Army Medical University and conducted in accordance with the Declaration of Helsinki. Informed consent for the use of all samples were obtained from each patient.

## Supplementary information

Supplemental Figure and Table Legends

Figure. S1

Figure. S2

Figure. S3

Figure. S4

Figure. S5

Figure. S6

Figure. S7

Table. S1

Table. S2

Table. S3

Table. S4

Table. S5

Table. S6

## Data Availability

All data needed to evaluate the conclusions in the paper are present in the paper and/or the Supplementary Materials. Additional data related to this paper may be requested from the authors.
