# Supplementary material for: Deep sequencing of circulating tumor DNA detects molecular residual disease and predicts recurrence in gastric cancer
Source: Cell Death Dis. 2020 May 11;11(5):346. doi: 10.1038/s41419-020-2531-z (PMC7214415; doi:10.1038/s41419-020-2531-z)
Supplement: Supplementary file 1 — Supplemental Figure and Table Legends [file 41419_2020_2531_MOESM1_ESM.docx]

**Supplemental Figure and Table Legends**

**Supplementary Figure 1. Mutation profile of primary gastric tumor.** Each column represents the primary tumor of one patient. For patients with more than one sequenced primary tumor, data from all tumors were combined and are shown in one column. Genes with mutations are shown in colored boxes. The numbers of mutations in each patient are shown at the top, and the percentage of patients who had mutations in each gene is shown on the left. Only genes that were mutated in more than two patients are shown on the plot.

**Supplementary Figure 2. Concordance of mutation profile between ctDNA and tumors.** Mutations identified only in ctDNA (red), tumor tissue (blue) and in both (orange) were shown, with genes shown on the bottom and patient ID shown on the left.

**Supplementary Figure 3. Mutation profile of treatment-naïve ctDNA.** All somatic mutations of pre-treatment ctDNA are shown. Clinical characteristics are shown on the bottom.

**Supplementary Figure 4. Mutation profile and changes for patient P038.** (A) Dynamic changes of variant allelic frequencies of mutations at different times after surgery for patient P038 are shown. Time of surgery, adjuvant chemotherapy, disease relapse, and death are indicated. (B) Observed mutations by targeted sequencing in different samples. Blue indicates mutation. (C) Clonal structure of primary tumor was inferred with WES data. Different colors indicate different clones, with light blue represents the most prevalent clone. Representative genes of several clones were shown with corresponding colors. Mutations observed in targeted sequencing were subclonal mutations, including *ERBB4*, *FCGR3A*, and *NOTCH4*. *GKN2* and *MED12* mutations were not observed in the WES data, which might due to relatively low WES coverage (138X) compared to targeted tumor sequencing (788X).

**Supplementary Figure 5. Dynamic changes of ctDNA in patients with disease relapse.** Maximum of variant allelic frequencies for mutations observed in ctDNA that changed at different times and are shown in each plot. Time of surgery, disease relapse, and death of patients are indicated. Chemotherapy with SOX regimen (grey box) and S-1 (yellow box) are shown.

**Supplementary Figure 6. Event-free survival of 19 cases with disease relapsed.** In 19 patients with relapsed disease, time to ctDNA positivity and radiology positive findings were compared, and ctDNA detection was observed before radiologic relapse.

**Supplementary Figure 7. Clonal changes during or after adjuvant chemotherapy.** Twenty-one new mutations, including *TP53*, *RB1*, *PIK3CA*, *ATR*, were detected from plasmas of six patients during or after the adjuvant chemotherapy.

**Supplementary Table 1.** Gene list.

**Supplementary Table 2.** Patient characteristics.

**Supplementary Table 3.** Somatic variations in tumor DNA.

**Supplementary Table 4.** clinical characteristics and pre-operative ctDNA status.

**Supplementary Table 5**. Clinicopathologic and molecular factors and their effects on Disease-Free-Survival and Overall-Survival by multivariate Cox proportional hazards regression.

**Supplementary Table 6.** Time-dependent sensitivity and specificity of post-operative ctDNA in predicting recurrence.
