# Supplementary figures and images for: Deep sequencing of circulating tumor DNA detects molecular residual disease and predicts recurrence in gastric cancer
Source: Cell Death Dis. 2020 May 11;11(5):346. doi: 10.1038/s41419-020-2531-z (PMC7214415; doi:10.1038/s41419-020-2531-z)

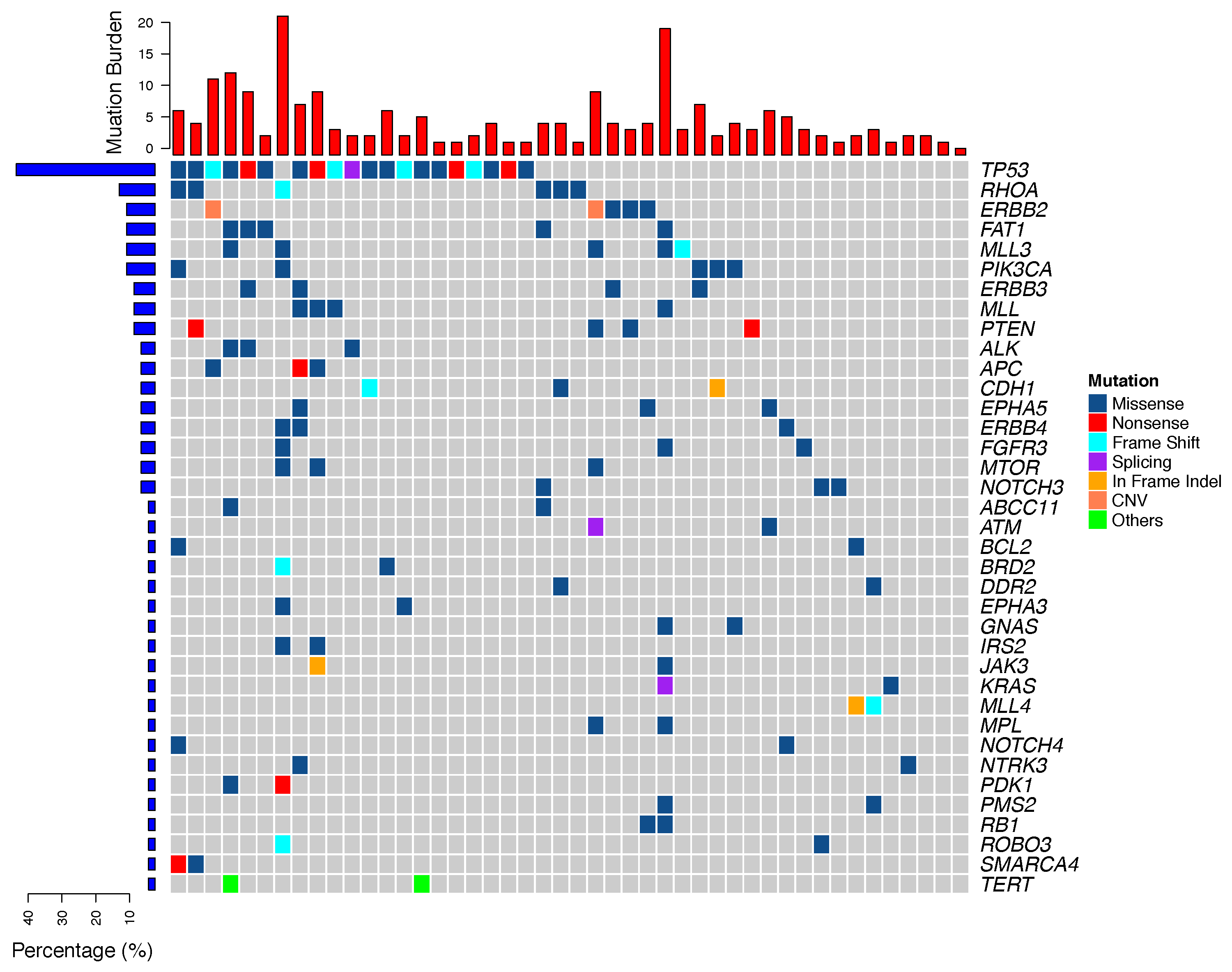

Supplement: Supplementary file 2 — Figure. S1 [file 41419_2020_2531_MOESM2_ESM.tif]

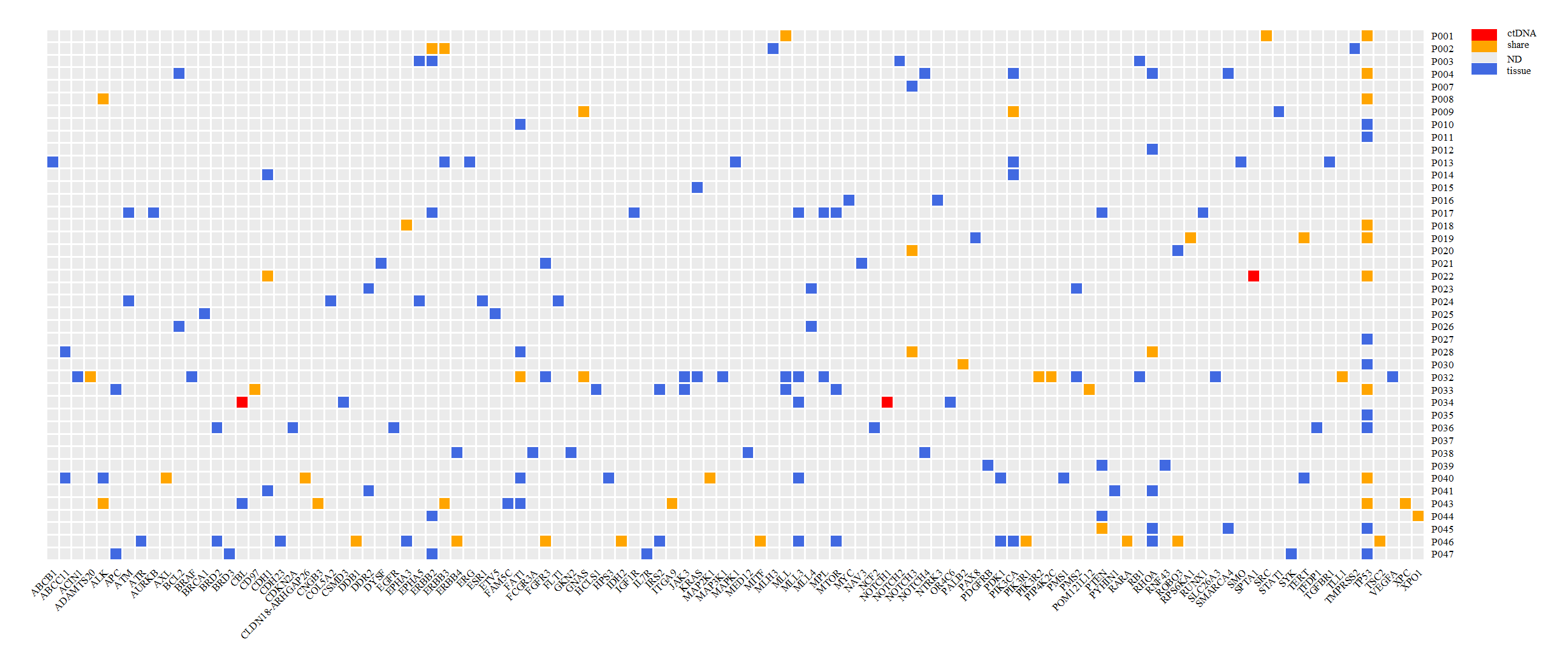

Supplement: Supplementary file 3 — Figure. S2 [file 41419_2020_2531_MOESM3_ESM.tif]

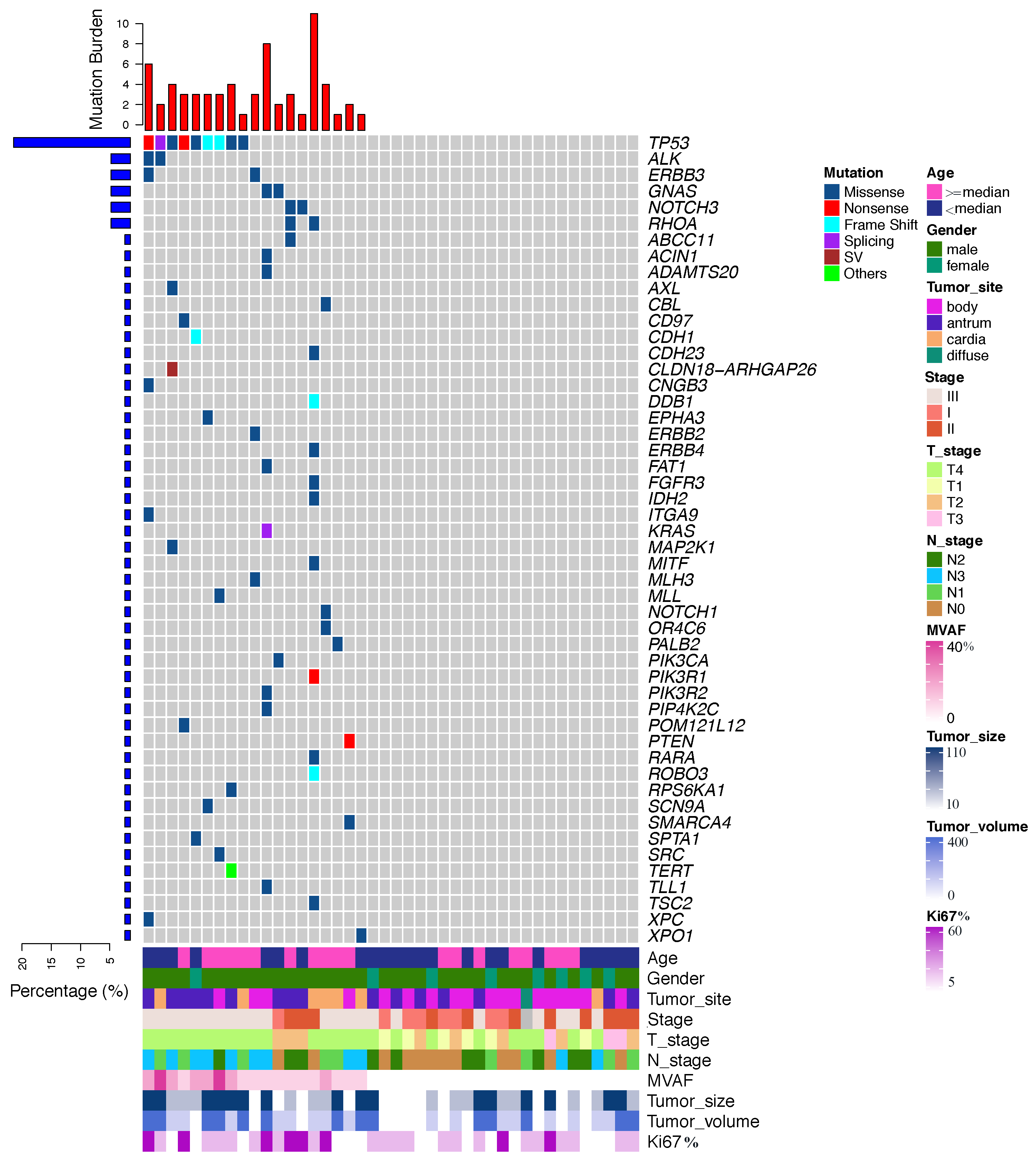

Supplement: Supplementary file 4 — Figure. S3 [file 41419_2020_2531_MOESM4_ESM.tif]

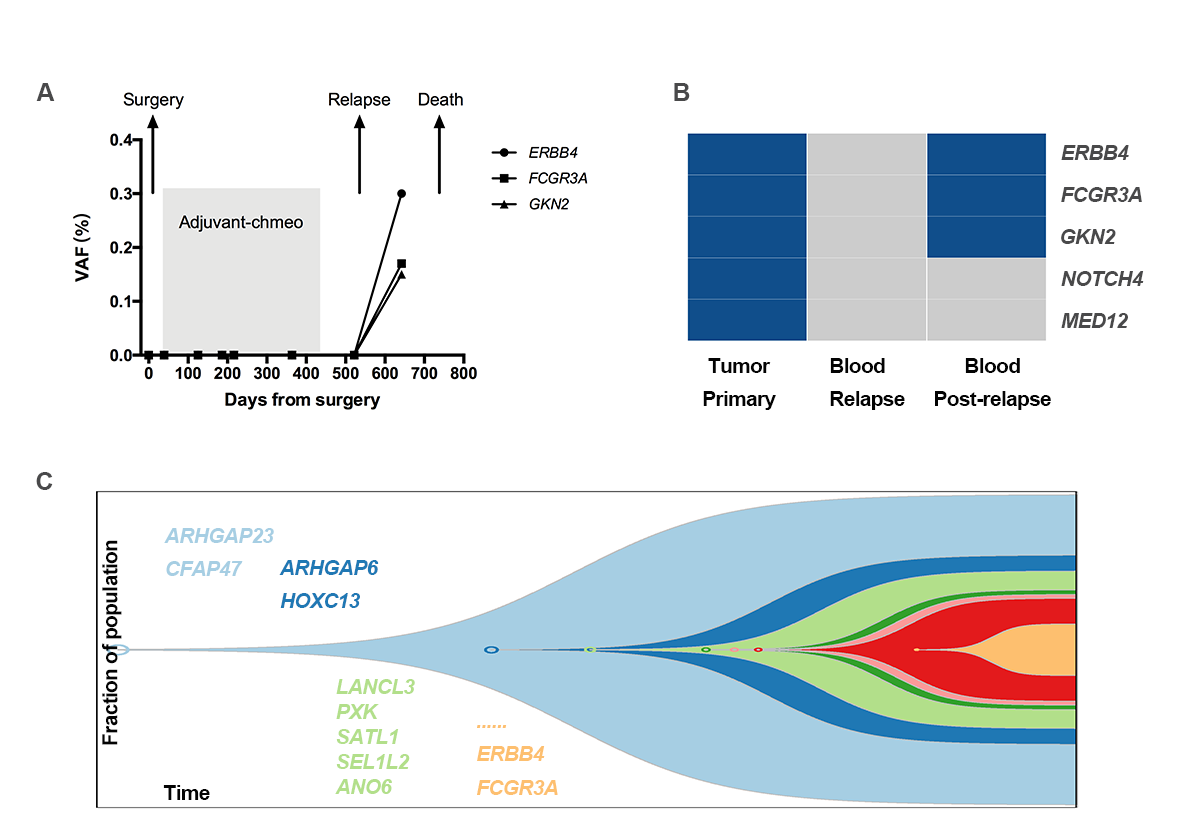

Supplement: Supplementary file 5 — Figure. S4 [file 41419_2020_2531_MOESM5_ESM.tif]

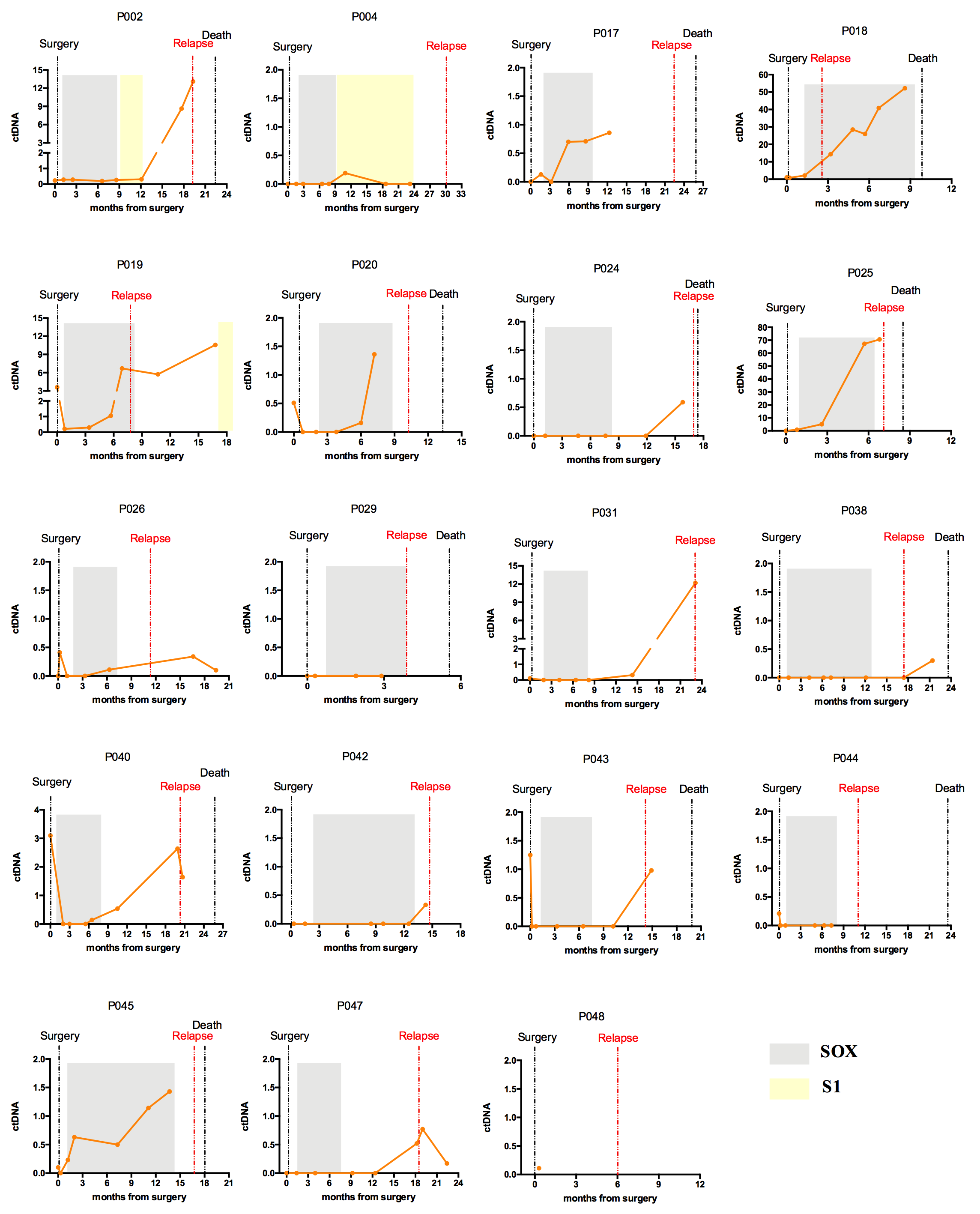

Supplement: Supplementary file 6 — Figure. S5 [file 41419_2020_2531_MOESM6_ESM.tif]

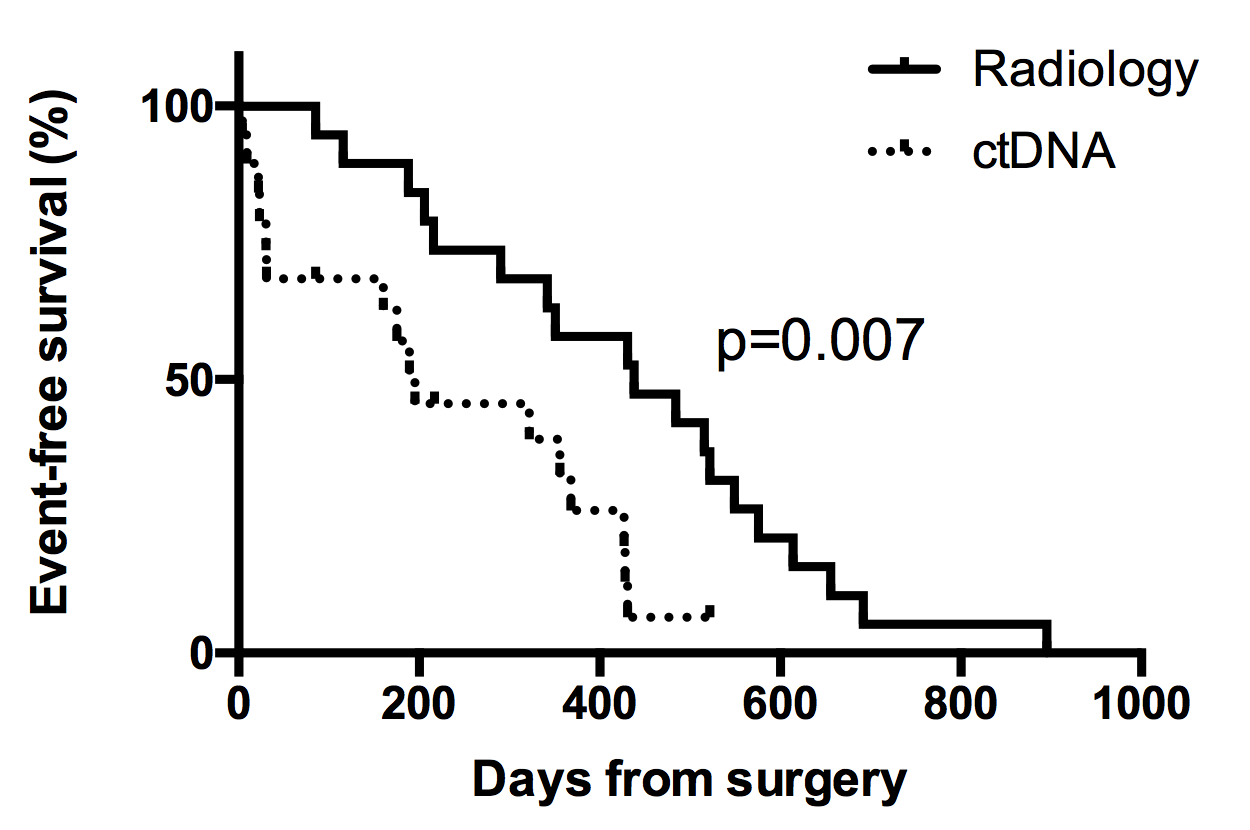

Supplement: Supplementary file 7 — Figure. S6 [file 41419_2020_2531_MOESM7_ESM.tif]

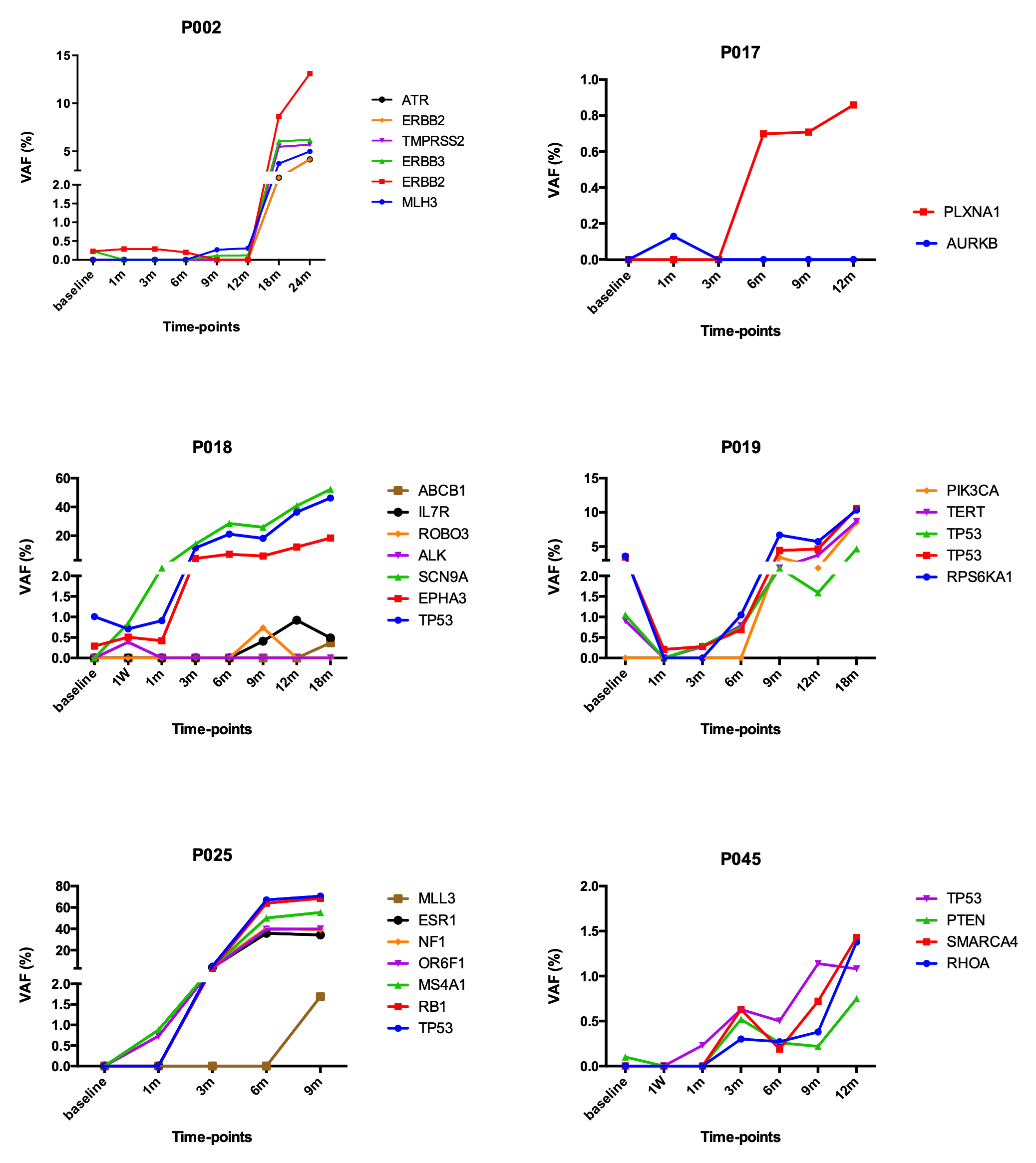

Supplement: Supplementary file 8 — Figure. S7 [file 41419_2020_2531_MOESM8_ESM.tif]
